# Study of the effect of health education based on the transtheoretical model in increasing the knowledge of the population covered by shaft Ahmadsargorab Healthcare Center to prevent drowning in 2017

**Published:** 2019-07

**Authors:** Nassibe Farmani Ghasabe

**Affiliations:** ^*a*^Student Research Committee of Public Health School, Guilan University of Medical Sciences, Rasht, Iran.

## Abstract

**Background:**

Guilan province undergoes a significant number of drowning annually which is the second common cause of death after traffic accidents. So far, some efforts have been made to this end, but using health education models has been neglected in the previous studies. Therefore, we aimed to measure the impact of health education based on the transtheoretical model in increasing the knowledge of the population in the current study.

**Methods:**

In this semi-experimental research, 550 households covered by Shaft Ahmadsargorab healthcare center participated in the study in March 2017. Initially, the knowledge level of 100 people was measured in a cross-sectional study, which was predicted to be low. After evaluating all structures of the trans-theoretical model, a pre-test was done in the total population, and the healthcare team trained the target group by a variety of teaching methods. A post-test was performed, and the results were analyzed using SPSS software.

**Results:**

The mean primary knowledge score of the population was 0.30 at the beginning of the study. The structures of the trans-theoretical model included change stages, 0.32, change process, 0.33, decision-making balance, 0.29, self-efficacy, 0.39. After doing intervention, the pre-test results changed as follows: the mean score of population knowledge became 0.85, and the structures of the trans-theoretical model changed). The number of failed drowning accidents-related reports in March 2018 compared to last year decreased by 0.60, and no successful drowning was recorded in the study year.

**Conclusions:**

This study showed using health education models plays an important role in promoting population knowledge and preventing drowning. The results of this study can be generalized to other populations. It is hoped that this study be a starting point for paying more attention to the use of health education models to prevent drowning and promote the health of our society.

**Keywords:**

Transtheoretical Model, Drowning, Ahmadsargorab

